# 

**Published:** 1930

**Authors:** 


### CONTENTS.

*Page*

**Observations on Epilepsy.** By J. V. Blachford, C.B.E., M.D. .. 169

**Five Years' Experience of Surgery in Exophthalmic Goitre.** By A. Rendle Short, B.Sc., M.D., B.S., F.R.C.S. .. .. .. 185

**The Treatment of Graves' Disease.** By C. E. K. Herapath, M.C., M.D. .. .. .. .. .. .. .. .. .. 193

**Changes in the Spleen in Acute Pyogenic Infections.** By T. F. R. Hewer, M.D. .. .. .. .. .. .. .. 197

**A Case of von Recklinghausen's Disease (Multiple Neuro-fibromata) with Spontaneous Fractures.** By L. P. Ashton 219

**The Origin and Significance of Tuberculosis of the Tracheo-Bronchial Lymph Nodes of the Lung.** By L. R. Jordan .. 225

**Reviews of Books** .. .. .. .. .. .. .. .. 239

**Editorial Notes** .. .. .. .. .. .. .. .. 248

**The Medical Library of the University of Bristol** .. .. 252

**Local Medical Notes** .. .. .. .. .. .. .. .. 254

